# Smooth Sensor Motion Planning for Robotic Cyber Physical Social Sensing (CPSS)

**DOI:** 10.3390/s17020393

**Published:** 2017-02-17

**Authors:** Hong Tang, Liangzhi Li, Nanfeng Xiao

**Affiliations:** 1 School of Computer Science & Engineering, South China University of Technology, Guangzhou 510641, China; tang.hong@mail.scut.edu.cn (H.T.); xiaonf@scut.edu.cn (N.X.); 2 Department of Information and Electronic Engineering, Muroran Institute of Technology, Muroran 050-0071, Japan

**Keywords:** sensors, robotic, binocular stereo vision, robotic sensing, trajectory planning, cyber physical social sensing (CPSS)

## Abstract

Although many researchers have begun to study the area of Cyber Physical Social Sensing (CPSS), few are focused on robotic sensors. We successfully utilize robots in CPSS, and propose a sensor trajectory planning method in this paper. Trajectory planning is a fundamental problem in mobile robotics. However, traditional methods are not suited for robotic sensors, because of their low efficiency, instability, and non-smooth-generated paths. This paper adopts an optimizing function to generate several intermediate points and regress these discrete points to a quintic polynomial which can output a smooth trajectory for the robotic sensor. Simulations demonstrate that our approach is robust and efficient, and can be well applied in the CPSS field.

## 1. Introduction

**Figure 1:**
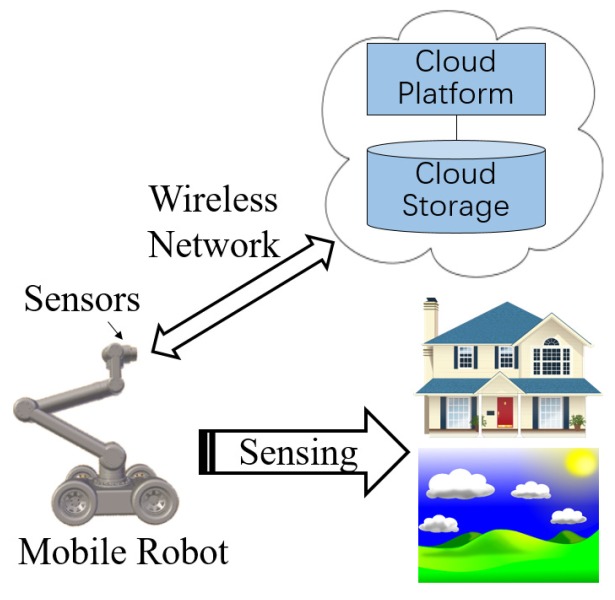
Mobile robotic sensing for Cyber Physical Social Sensing (CPSS).

With the huge developments in sensing and network technologies, Cyber Physical Social Sensing (CPSS) has attracted the attention of many researchers [1,2,3,4]. Although much research has been proposed to advance the field of CPSS, little research has focused on social robotic sensing. We successfully utilize robots in the CPSS area, and propose an effective robotic sensing method in the paper. As shown in Figure 1, we use some robots equipped with specially-designed eye-in-hand sensors to explore the world, and share the information among all robots using a wireless network and cloud platform. To move sensors accurately and smoothly, robots need to calculate their trajectory. Traditional methods, however, cannot be applied directly to robotic sensing in CPSS, mainly because the existing trajectory planning methods are not designed for sensing tasks. Historically, most trajectory planning methods are not suitable for eye-in-hand sensors because of their low efficiency due to the extra calculation of inverse kinematics, instability coming from inadequacy of the traditional methods with sensor performance optimization, and non-smooth-generated paths. To solve these problems, we propose a novel trajectory planning method to improve the sensing performance in CPSS.

Trajectory planning is a fundamental problem in robotics. Because of its limitations, both the velocity and acceleration of robotic drivers cannot achieve the ideal level. Robots are multi-variable and have highly nonlinear complex systems. It is extremely difficult to obtain a smooth trajectory to simultaneously meet the requirements of velocity, acceleration, and jerk. Some trajectory planning methods (e.g., C-space [5] and preprocessing algorithms) can find a smooth trajectory that satisfies the kinematic limits [6,7]. Most of these traditional methods, however, are focused merely on time and jerk optimization [8,9,10], and visual information is not used. In the past decade, significant progress has been made in machine vision technology [11], and it has been applied to trajectory planning methods to improve planning performance. Li et al. [12] adopted vision-guided robot control to build a visual feedback system for real-time measurement of the end-effector and the joint position. Among all machine vision methods, classic binocular stereo-vision—which captures the same image from two angles using two cameras—is used most widely for its simple configuration and high reliability, and this method is adopted as the visual system in this paper. Using the stereo-matched algorithm [11], the disparity between two images can be calculated. Following this calculation, the three-dimensional (3D) position and orientation of the objects can be obtained using the camera calibration technique, which illustrates the mapping relationship between the pixels in the digital image and the 3D position in the world coordinate system.

Trajectory planning methods use a series of transformation matrices [13,14,15] to obtain the position of each joint in one robot. When the reverse kinematics method is used to calculate the joint angle for a given manipulator position, the solution trajectory of relevant joints is usually not distinct. Therefore, the optimization objective must be determined to arrive at the optimal trajectory [16,17]. Another problem results form the joint positioning errors caused by weight distribution, load change, vibration, mechanical friction, and recoil, making it difficult to obtain an accurate robotic dynamics model in real-world applications. Instead of providing the complete trajectory (which would have some deviation in the actual robotic motion), our approach is to provide the next position that can be reached at the next time unit. We believe that it is not necessary to get an accurate rotation angle for each joint, and instead focus on how the end-effector of the robot reaches the object continually and smoothly to achieve better sensing performance. In practical applications, the working precision of the robot is confined by such factors as manipulator limitations and working environments, which cause various errors in the sensor’s motion. We use binocular stereo-vision to rectify these motion errors. Both velocity and acceleration of the joint must be continuous, and therefore, the proposed method introduces jerk restriction to avoid vibration and reduce mechanical wear. To make a smooth motion path of the equipped sensor, we adopt an optimizing function to generate several intermediate points and regress these discrete points to a quintic polynomial, which ultimately outputs a smooth trajectory for the sensor.

## 2. System Overview

Generally, the robotic motion trajectory is described in Cartesian space or joint space. The trajectory represented in the joint angle space, however, offers several advantages [18]. First, the trajectory directly generated by the angle rotation avoids a lot of forward and inverse kinematics calculation—in particular for real-time applications. Second, the trajectory represented in Cartesian space will eventually be converted into the joint coordinates. If the trajectory is generated directly in the joint space, it is clear that the computation time can be reduced. To improve position accuracy, a visual sensor is used to compensate for errors and correct the trajectory.

**Figure 2:**
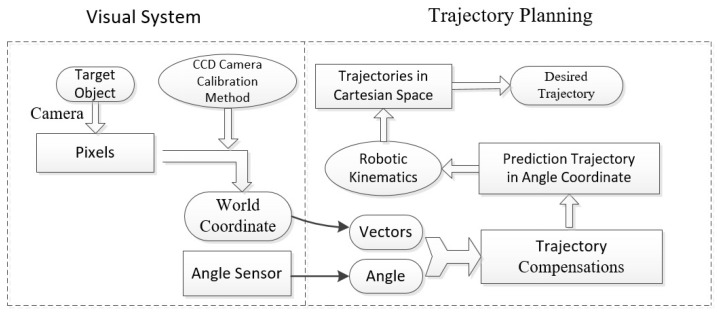
Schematic of the proposed trajectory planning method.

Due to the accumulated error, the manipulator cannot reach the target to be processed when the manipulator is running on a predetermined trajectory. In order to improve the accuracy, we use the binocular stereo visual sensor to compensate and correct the trajectory. Therefore, the schematic of the trajectory planning method proposed in this paper is divided into a visual module and trajectory planning module, and Figure 2 shows the schematic of the trajectory planning method with the binocular stereo visual sensor. In our method, the planning trajectory is first generated according to the initial states and the end states of the manipulator. When the manipulator is running, the trajectory of the manipulator is corrected by acquiring the joint angles of the current joints and the positions of the distal end of the manipulator, which can improve the grasping accuracy of the manipulator. In order to compensate for the trajectory, we need to measure some parameters, in which the joint angles are obtained by the angle sensors, and the end position of the manipulator needs to use the binocular stereo visual sensor and the stereo vision algorithm. The stereoscopic vision algorithm can give the mapping from the pixel coordinates to the spatial coordinates. This provides us a great convenience to calculate the trajectory compensation values. Therefore, it can be seen that the binocular stereo visual sensor plays an great important role in improving the grasping progress of the target to be processed. After obtaining the trajectory of the angular space, the trajectory in the Cartesian space can be obtained by the forward kinematics of the manipulator.

After getting the trajectory represented in joint angle space, we can transform it into Cartesian space by robotic forward kinematics.

### 2.1. Binocular Vision Sensor

**Figure 3:**
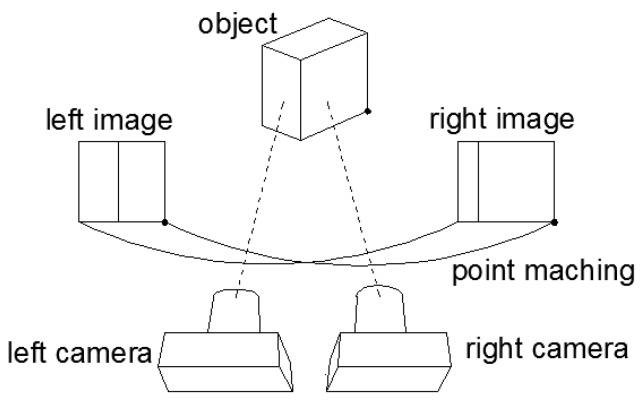
Visual sensor and binocular positioning.

The proposed vision method is shown in Figure 3, which illustrates how the binocular vision sensor is used to improve the manipulator’s operation accuracy. Many other machine vision methods have been applied in trajectory planning. Classical binocular stereovision, however, is still widely used for its simplicity and effectiveness. This method uses two vision sensors to obtain different images of the same object from different angles. Then, the Cartesian coordinate of the target can be obtained by finding the difference between the two visual images.

Currently, RGB-Depth sensors and optical fiber sensors [19] are used widely to measure the Cartesian coordinate of the object. If, however, some obstacles appear between the sensor and the object, these sensors may fail to get the location and the orientation of the object. Further, additional computational costs are required to obtain the absolute object coordinates for trajectory planning. Because relative position is more important than absolute position to control the mobile robot and the equipped sensor, traditional binocular stereo-vision is adopted in the proposed method.

### 2.2. Testing Platform

The proposed system was tested in a simulation environment called Robot Operating System (ROS). ROS is a software framework for robotic research and development, and has been a mainstream robotic simulation platform. This system integrates hardware abstraction, device drivers, libraries, visualizers, message-passing, package management, and many other convenient functions. The UR5 robot is a robot with six degrees of freedom (6-DOF). In our experiments, it is equipped with a mobile chassis and a binocular stereo vision sensor to conduct the performance evaluations.

## 3. Binocular Stereo Vision Sensor System

To generate the transform vector that directly maps the end-effector to the target, the mapping relationship between the space positions and the pixel locations in the camera plane is required. Considering the influence of lens distortion, the transformation matrix from the camera coordinate to the world coordinate [14] can be expressed by a homogeneous transformation matrix, per Equation (1):

(1) $$Z_{c}{\left[u\;\;\;\;\;\;\;v\;\;\;\;\;\;\;1\right]}^{T}=M^{*}P_{W}$$

where $\left(u,v\right)$ is a pixel of a point in the camera image plane, its homogeneous coordinates are ${\left(u,v,1\right)}^{T}$; $P_{W}$ is the world coordinate of a point, its homogeneous coordinates are described as ${\left[X_{w}\;Y_{w}\;Z_{w}\;1\right]}^{T}$. The transformation matrix $M^{*}$ can be described as follows:

(2) $$M^{*}\;=\;\;\left[\begin{array}{c} f/dx\;\;\;\;\;\;\;\;\;\;\;\;0\;\;\;\;\;\;\;\;\;\;\;\;\;\;u_{0}\\ \;\;\;\;\;\;\;\;0\;\;\;\;\;\;\;\;\;\;\;\;\;f/dy\;\;\;\;\;v_{0}\;\;\;\\ \;\;\;\;\;\;\;\;0\;\;\;\;\;\;\;\;\;\;\;\;\;\;\;\;\;\;\;\;\;0\;\;\;\;\;\;\;\;\;\;\;\;\;\;\;\;1\;\;\;\;\;\;\;\;\;\;\; \end{array}\right]\left[\begin{array}{c} f\;\;\;\;\;\;0\;\;\;\;\;\;0\;\;\;\;\;\;0\\ 0\;\;\;\;\;\;f\;\;\;\;\;\;0\;\;\;\;\;\;0\\ 0\;\;\;\;\;\;\;0\;\;\;\;\;\;1\;\;\;\;\;\;0\;\;\;\; \end{array}\right]\left[\begin{array}{c} R_{3\times 3}\;\;\;\;\;\;t_{3\times 1}\\ \;0_{1\times 3}\;\;\;\;\;\;\;\;1 \end{array}\right]$$

**Figure 4:**
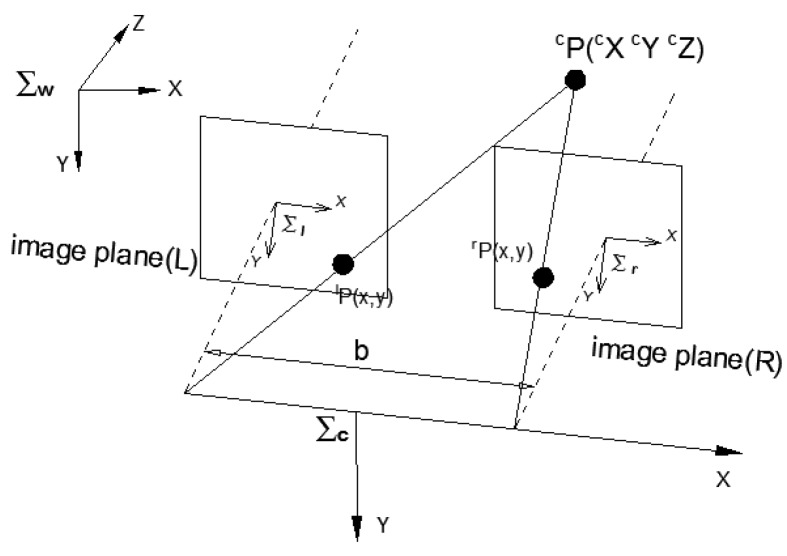
Binocular stereo sensor.

The matrix $M^{*}$ can be obtained easily by Zhang’s calibrating method [20]. In Equation (2), $\left(u_{0},v_{0}\right)$ is the origin coordinate of the physical coordinates in camera image plane. dx and dy are the length and the width of a pixel, respectively. $R_{3\times 3}$ and $t_{3\times 1}$ are respectively the rotation matrix and the translation vector of the camera coordinate frame to the world coordinate system. In order to get the world coordinate of the point, the homogeneous coordinates of the point $\left[X_{c}\;Y_{c}\;Z_{c}\;1\right]$ in camera coordinate system is needed. The coordinates $\left[X_{c}\;Y_{c}\;Z_{c}\;1\right]$ is obtained by binocular stereo vision, which provides additional information about the objects and environments through the left and right cameras. If we obtain the perspective difference between the left and right camera images, we then can calculate the coordinates of the target point. The parallax principle of the binocular stereovision is shown in Figure 4; ${}^{l}p$ and ${}^{r}p$ are the points at which the target point ${}^{c}p$ is projected on the left and right camera planes, respectively. If *b* is the distance between the optical centers of the left and right cameras, the coordinates of ${}^{c}p$ in the left and right camera planes are $\left(x_{left}=f\;X_{c}/Z_{c},y_{left}=f\;Y_{c}/Z_{c}\right)$ and $(x_{right}=f(X_{c}$$-b)/Z_{c},y_{right}=f\;Y_{c}/Z_{c})$.

The visual disparity between the left and right image planes then can be obtained by $disp\;=\;x_{left}-x_{right}$. Finally, the coordinates of point ${}^{c}p$ in the camera coordinate system can be calculated [9] by Equation (3):

(3) $$X_{c}\;\;\;\;=\frac{b\cdot x_{left}}{disp},\;\;\;Y_{c}\;\;\;\;\;\;\;\;=\frac{b\cdot y_{left}}{disp}\;\;\;,\;\;\;\;Z_{c}\;\;\;\;\;\;\;=\frac{b\cdot f\;}{disp}$$

Equation (3) shows the mathematical model of the transformation from the pixel to the Cartesian coordinate.

## 4. Trajectory Planning For Binocular Stereo Sensors

Generally, a trajectory is obtained by some calculations pertaining to the initial states and the final states (e.g., position, velocity, and acceleration) of the joints in the Cartesian coordinates. The points on the trajectory must then be mapped to a set of joint angles by inverse kinematics calculation. In fact, the robotic motion is actually the rotary movement of each of joints. Therefore, the trajectory represented in the joint coordinate system can describe the robotic motion more directly.

### 4.1. Joint Space-Based Trajectory Planning

A smooth interpolation function is required to obtain a satisfactory joint trajectory connecting the initial joint angles and the final joint angles. Considering the constraints, a five-order interpolation function [9] is used to calculate the robotic trajectory. In this calculation, $\theta \left(t\right)$ is defined as a joint trajectory function that describes the relationship between the joint angle and time; $t_{b}$, $\theta_{b}$, ${\dot{\theta }}_{b}$, and ${\ddot{\theta }}_{b}$ represent the initial state of time, joint angle, angular velocity, and angular acceleration, respectively; and $t_{f}$, $\theta_{f}$, ${\dot{\theta }}_{f}$, and ${\ddot{\theta }}_{f}$ represent the final state of time, joint angle, angular velocity, and angular acceleration, respectively. The five-order interpolation function can be described as follows:

(4) $$s\left(t\right)=\ell_{0}+\ell_{1}t+\ell_{2}t^{2}+\ell_{3}t^{3}+\ell_{4}t^{4}+\ell_{5}t^{5}\;\;\;$$

Let $T_{f}=t_{f}-t_{b}$. $T_{p}$—which is determined by the controller of the manipulator—is the trajectory sampling period, then the sampling number num is $\frac{T_{f}}{T_{p}}$. Define *τ* as the sequence number of sample points, $\tau =\left(t-t_{b}\right)/T_{p}$, τ∈[0,num], then the trajectory can be represented by discrete sampling points as follows:

(5) $$\theta \left(\tau \right)=\theta_{b}+\left(\theta_{f}-\theta_{b}\right)s\left(\tau \right)$$

The first derivative and the second derivative of Equation (5) can be expressed as

(6) $$\dot{\theta }\left(\tau \right)=\left(\theta_{f}-\theta_{b}\right)\frac{\dot{s}\left(\tau \right)}{T_{p}}\;$$

(7) $$\ddot{\theta }\left(\tau \right)=\left(\theta_{f}-\theta_{b}\right)\frac{\ddot{s}\left(\tau \right)}{{T_{p}}^{2}}$$

In Equations (5)–(7), the initial and final states are known; let $\theta \left(t_{b}\right)=\theta_{b}$, $\dot{\theta }\left(t_{b}\right)={\dot{\theta }}_{b}$, $\ddot{\theta }\left(t_{b}\right)={\ddot{\theta }}_{b}$, $\theta \left(t_{f}\right)=\theta_{f}$, $\dot{\theta }\left(t_{f}\right)={\dot{\theta }}_{f}$, $\ddot{\theta }\left(t_{f}\right)={\ddot{\theta }}_{f}$, then putting the initial conditions and termination conditions into Equations (5)–(7), we can obtain

(8) $$\left\{\begin{array}{c} s\left(0\right)=0\\ \dot{s}\left(0\right)={\dot{\theta }}_{b}T_{p}/\left(\theta_{f}-\theta_{b}\right)\\ \ddot{s}\left(0\right)={\ddot{\theta }}_{b}{T_{p}}^{2}/\left(\theta_{f}-\theta_{b}\right) \end{array}\right.\;\;\;\;\;\;\;\;\;\;\;\;\;\;\;\;\;\;\;\;\;\;\;\;\;\;\;\;\left\{\begin{array}{c} s\left(num\right)=1\\ \dot{s}\left(num\right)={\dot{\theta }}_{f}T_{p}/\left(\theta_{f}-\theta_{b}\right)\\ \ddot{s}\left(num\right)={\ddot{\theta }}_{f}{T_{p}}^{2}/\left(\theta_{f}-\theta_{b}\right) \end{array}\right.$$

Present Equation (8) as matrix by Equation (4) and initial conditions, we can get the following:

(9) $$\underset{s}{\underset{︸}{\left(\begin{array}{c} s\left(0\right)\\ \dot{s}\left(0\right)\\ \ddot{s}\left(0\right)\\ s\left(num\right)\\ \dot{s}\left(num\right)\\ \ddot{s}\left(num\right) \end{array}\right)}}=\underset{M}{\underset{︸}{\left[\begin{array}{c} 1\;\;\;\;\;\;\;\;\;\;\;\;\;\;\;\;\;\;\;0\;\;\;\;\;\;\;\;\;\;\;\;\;\;\;\;\;\;\;\;\;0\;\;\;\;\;\;\;\;\;\;\;\;\;\;\;\;\;\;\;\;\;\;\;\;\;\;\;\;\;\;0\;\;\;\;\;\;\;\;\;\;\;\;\;\;\;\;\;\;\;\;\;\;\;\;\;\;\;\;\;\;\;\;\;\;0\;\;\;\;\;\;\;\;\;\;\;\;\;\;\;\;\;\;\;\;\;\;\;\;\;\;\;\;\;\;\;\;\;\;\;\;\;\;\;0\\ 0\;\;\;\;\;\;\;\;\;\;\;\;\;\;\;\;\;\;1\;\;\;\;\;\;\;\;\;\;\;\;\;\;\;\;\;\;\;\;\;\;0\;\;\;\;\;\;\;\;\;\;\;\;\;\;\;\;\;\;\;\;\;\;\;\;\;\;\;\;\;\;0\;\;\;\;\;\;\;\;\;\;\;\;\;\;\;\;\;\;\;\;\;\;\;\;\;\;\;\;\;\;\;\;\;\;0\;\;\;\;\;\;\;\;\;\;\;\;\;\;\;\;\;\;\;\;\;\;\;\;\;\;\;\;\;\;\;\;\;\;\;\;\;\;\;0\\ 0\;\;\;\;\;\;\;\;\;\;\;\;\;\;\;\;\;\;0\;\;\;\;\;\;\;\;\;\;\;\;\;\;\;\;\;\;\;\;\;\;2\;\;\;\;\;\;\;\;\;\;\;\;\;\;\;\;\;\;\;\;\;\;\;\;\;\;\;\;\;\;0\;\;\;\;\;\;\;\;\;\;\;\;\;\;\;\;\;\;\;\;\;\;\;\;\;\;\;\;\;\;\;\;\;\;0\;\;\;\;\;\;\;\;\;\;\;\;\;\;\;\;\;\;\;\;\;\;\;\;\;\;\;\;\;\;\;\;\;\;\;\;\;\;\;0\\ 1\;\;\;\;\;\;\;\;\;\;\;\;\;\;\;\frac{T_{f}}{T_{p}}\;\;\;\;\;\;\;\;\;\;\;\;{\left(\frac{T_{f}}{T_{p}}\right)}^{2}\;\;\;\;\;\;\;\;\;\;\;{\left(\frac{T_{f}}{T_{p}}\right)}^{3}\;\;\;\;\;\;\;\;\;\;\;\;\;{\left(\frac{T_{f}}{T_{p}}\right)}^{4}\;\;\;\;\;\;\;\;\;\;\;\;\;\;\;\;\;\;\;\;{\left(\frac{T_{f}}{T_{p}}\right)}^{5}\\ 0\;\;\;\;\;\;\;\;\;\;\;\;\;\;\;\;\;\;1\;\;\;\;\;\;\;\;\;\;\;\;\;2\frac{T_{f}}{T_{p}}\;\;\;\;\;\;\;\;\;\;\;\;\;\;\;3\;{\left(\frac{T_{f}}{T_{p}}\right)}^{2}\;\;\;\;\;\;4\;{\left(\frac{T_{f}}{T_{p}}\right)}^{3}\;\;\;\;\;\;\;\;\;\;5{\left(\frac{T_{f}}{T_{p}}\right)}^{4}\\ 0\;\;\;\;\;\;\;\;\;\;\;\;\;\;\;\;\;\;0\;\;\;\;\;\;\;\;\;\;\;\;\;\;\;\;\;\;\;\;\;2\;\;\;\;\;\;\;\;\;\;\;\;\;\;\;\;\;\;\;\;\;\;\;6\frac{T_{f}}{T_{p}}\;\;\;\;\;\;\;\;\;\;\;\;\;\;\;12\;{\left(\frac{T_{f}}{T_{p}}\right)}^{2}\;\;\;\;\;\;\;20{\left(\frac{T_{f}}{T_{p}}\right)}^{3}\; \end{array}\right]}}\underset{\ell }{\underset{︸}{\left(\begin{array}{c} \ell_{0}\\ \ell_{1}\\ \ell_{2}\\ \ell_{3}\\ \ell_{4}\\ \ell_{5} \end{array}\right)}}\;\;\;\;\;$$

So, vector *ℓ* can be expressed as follows:

(10) $$\;\;\ell =M^{-1}{\left(0\;\;\;\;\;\;\;\;\;\;\;\frac{{\dot{\theta }}_{b}T_{p}}{\theta_{f}-\theta_{b}}\;\;\;\;\;\;\;\;\;\;\;\;\frac{{\ddot{\theta }}_{b}{T_{p}}^{2}}{\theta_{f}-\theta_{b}}\;\;\;\;\;\;\;\;\;1\;\;\;\;\;\;\;\;\;\frac{{\dot{\theta }}_{f}T_{p}}{\theta_{f}-\theta_{b}}\;\;\;\;\;\;\;\;\;\frac{{\ddot{\theta }}_{f}{T_{p}}^{2}}{\theta_{f}-\theta_{b}}\right)}^{T}$$

Then, the trajectory in joint coordinate can be obtained by Equations (5) and (10).

(11) $$\theta \left(\tau \right)=\theta_{b}+\left(\theta_{f}-\theta_{b}\right)M^{-1}{\left(0\;\;\;\;\;\;\;\;\;\;\;\frac{{\dot{\theta }}_{b}T_{p}}{\theta_{f}-\theta_{b}}\;\;\;\;\;\;\;\;\;\;\;\;\frac{{\ddot{\theta }}_{b}{T_{p}}^{2}}{\theta_{f}-\theta_{b}}\;\;\;\;\;\;\;\;\;1\;\;\;\;\;\;\;\;\;\frac{{\dot{\theta }}_{f}T_{p}}{\theta_{f}-\theta_{b}}\;\;\;\;\;\;\;\;\;\frac{{\ddot{\theta }}_{f}{T_{p}}^{2}}{\theta_{f}-\theta_{b}}\right)}^{T}\left[1\;\;\;\tau \;\;\tau^{2}\;\;\;\tau^{3}\;\;\;\tau^{4}\;\;\;\tau^{5}\;\right]$$

The relationship between the trajectories represented in the Cartesian space and the joint space can be expressed as follows:

(12) $$X\left(t\right)=f\left(\theta \left(t\right)\right)$$

where $X\left(t\right)\;$ and $\theta \left(t\right)$ are the trajectories in the Cartesian space and the joint space, respectively. The velocity is a constraint factor that needs to be considered, and the mapping relationship can be obtained by a derivative of Equation (12) as follows:

(13) $$\dot{X}\left(t\right)=\frac{\partial f\left(\theta \left(t\right)\right)}{\partial t}=J\left(\theta \left(t\right)\right)\;\;\;\;\;\;\dot{\theta }\left(t\right)$$

### 4.2. Coordinate Transformation

The joint positions relative to the base of the manipulator, as well as the positional relationship between the end-effector and the target object are required to measure the target position. In the proposed method, the joint coordinates in the Cartesian space are used in each iteration, and the coordinates in the angular space are used to calculate the joint position in each iteration. The Cartesian coordinates of the joints are obtained by using the following forward kinematics equation. The 6-DOF robot is represented and modeled by the D-H method [14,15], the transformation matrix of link j+1 to link *j* is ${}_{j+1}^{j}A$, and the forward kinematics equations of the 6-DOF robot can be expressed as

(14) $$T={}_{K}^{0}A={}_{1}^{0}A\;\;\;\;{}_{2}^{1}A\cdots \;{}_{j+1}^{j}A\cdots$$

**Figure 5:**
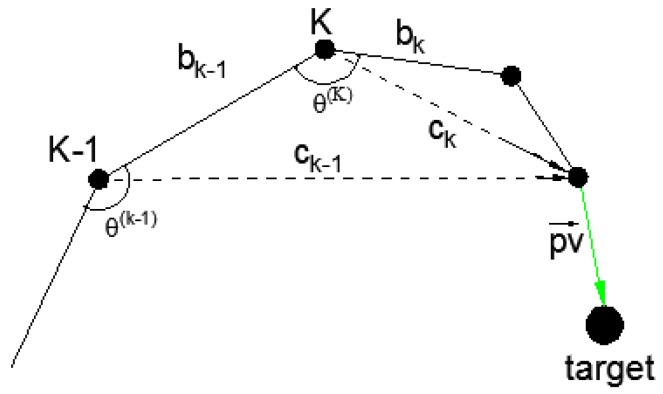
Schematic of the proposed method.

During the operation of the robot, the relative position of joint *j* can be obtained by using ${}_{j}^{0}A$. In the proposed method, ***b_k_***, ***c_k_***, and ***pv*** shown in Figure 5 need to be calculated using the transformation matrix and the input of the binocular stereovision sensor. If the D-H parameters of the robot are determined, the Cartesian coordinate of the end-effector in the base coordinate system can be calculated by using forward kinematics equations T defined in Equation (14).

### 4.3. Joint State

Generally, traditional trajectory planning methods merely concentrate on the calculation of the end effector position using the joint angles. These methods calculate the position from joint angle readings through the direct kinematics model to estimate the actual position. However in most instances, these positions are not actually reached due to the mechanical error. A novel trajectory planning method is proposed in the paper to analyze current joint states. A schematic of the proposed method is shown in Figure 5. Suppose *k* is the joint ordinal; ***b*${}_{k}$** (*k* = 1, 2, ..., n−1) represents the vector of the link; ***pv*** is an approach vector from the end-effector to the target; and ***c*${}_{k}$** denotes the vector from the *k*th joint to the end-effector. ***c*${}_{k}$** can be expressed as

(15) $$c_{k}=b_{k}+c_{k+1}$$

where the penultimate joint ${c_{n}}_{-1}={b_{n}}_{-1}$.

The D-H parameters and joint angles are required to obtain the vectors shown in Figure 5. The joint angles can be obtained by the joint angle sensors. The Cartesian coordinates of the end-effector and target can be measured using the visual sensors. In Section 4.1, $T_{p}$ is defined as the sampling period, num is the sampling times, and $t_{b}$ and $t_{f}$ represent the start and final time, respectively. So, the trajectory planning problem can be described as follows:

(16) $$\theta_{b}^{\left(k\right)}=\theta^{\left(k\right)}\left(0\right);\;\;\;\;\omega_{b}^{\left(k\right)}={\dot{\theta }}^{\left(k\right)}\left(0\right);\;\;\;\;\alpha_{b}^{\left(k\right)}={\ddot{\theta }}^{\left(k\right)}\left(0\right)$$

(17) $$\begin{array}{c} \theta_{f}^{\left(k\right)}=\theta^{\left(k\right)}\left(num\cdot T_{p}\right);\;\;\;\;\;\omega_{f}^{\left(k\right)}={\dot{\theta }}^{\left(k\right)}\left(num\cdot T_{p}\right);\;\;\\ \;\;\alpha_{f}^{\left(k\right)}={\ddot{\theta }}^{\left(k\right)}\left(num\cdot T_{p}\right) \end{array}$$

(18) $$\omega^{\left(k\right)}\left(jT_{p}\right)\leq \omega_{\max}^{\left(k\right)}\;\;\;and\;\;\;\;\alpha^{\left(k\right)}\left(jT_{p}\right)\leq \alpha_{\max}^{\left(k\right)},\;\;\;\;\;j\in \left[0,num\right]$$

(19) $$\left|\frac{\alpha^{\left(k\right)}\left(mT_{p}\right)-\alpha^{\left(k\right)}\left(nT_{p}\right)}{\left(m-n\right)T_{p}}\right|\leq j_{\max},\;\;\;\;\;\;0\leq m,n\leq num$$

If the second-order reciprocal of $\theta^{\left(k\right)}$ is continuous and satisfies Equations (16)–(19), then $\theta^{\left(k\right)}$ can be used as a trajectory solution that minimizes acceleration, shock acceleration, or run time. In the proposed method, a robot with *K* joints has $2^{K}$ motion patterns. The solved trajectory does not need to satisfy all of the previously mentioned optimization goals. In practice, the number of joints of the robot is usually small. For example, *K* is six in our simulation. Moreover, ***c*${}_{k}$** is a major factor to control the rotation of the joint. Given a state of the manipulator, some ***c*${}_{k}$** are important to reduce the length of ***pv***, whereas other ***c*${}_{k}$** can affect the orientation of ***pv***. How ***c*${}_{k}$** affects ***pv*** can be determined by the angle between ***c*${}_{k}$** and ***pv***. If, for example, ***c*${}_{k}$** is almost perpendicular to ***pv***, its main function is to change the length of ***pv*** so it can be used to reduce the distance from the end-effector to the target. On the contrary, if ***c*${}_{k}$** is almost parallel to ***pv***, it is used to change the orientation of ***pv***. As shown in Figure 5, ***c${}_{k}$${}_{-}$${}_{1}$*** is the main factor to reduce the length of ***pv***, whereas ***c*${}_{k}$** is more effective when it is used to change the orientation of ***pv***. The angle between ***c*${}_{k}$** and ***pv*** can be obtained by

(20) $$cos\vartheta^{\left(k\right)}=\frac{pv\cdot c_{k}}{∥pv∥\cdot ∥c_{k}∥}$$

Next, the angle increment of each joint $\Delta \theta^{\left(k\right)}$ needs to be determined. The effect of the joint rotation on the ***pv*** can be expressed by $\sum \left(c_{k}\cdot sin\vartheta^{\left(k\right)}\right)$ and $\sum \left(c_{k}\cdot cos\vartheta^{\left(k\right)}\right)$, where $\sum \left(c_{k}\cdot sin\vartheta^{\left(k\right)}\right)$ represents the angle increment in the parallel orientation of ***pv***, and $\sum \left(c_{k}\cdot cos\vartheta^{\left(k\right)}\right)$ represents the angle increment in the perpendicular orientation of ***pv***. To simplify the computation, ***tr*${}^{\left(k\right)}$** is defined in Equation (21) as follows:

(21) $$tr^{\left(k\right)}={\left[\sum \left(∥c_{k}∥\cdot \Delta \theta^{\left(k\right)}\cdot sin\vartheta^{\left(k\right)}\right),\;\;\;\;\sum \left(∥c_{k}∥\cdot \Delta \theta^{\left(k\right)}\cdot cos\vartheta^{\left(k\right)}\right)\right]}^{T}$$

In Equation (21), ***pv***, ***c*${}_{k}$**, and $\vartheta^{\left(k\right)}$ can be calculated. ***tr*${}^{\left(k\right)}$** is used to construct the optimization function, as shown in Equation (22). Then, determining the next position of the joint can be transformed into finding an appropriate $\Delta \theta^{\left(k\right)}$ such that it satisfies Equation (22).

(22) $$\Delta \theta^{\left(k\right)}=arg\;\;\;\;max\left(tr^{\left(k\right)}\cdot pv\right)$$

Considering the influence of the restrictive conditions of speed, acceleration, and jerk, some important variables must be defined. Because the motion trajectory of the end-effector is affected by the rotation of the joints, two important factors will be considered when correcting the trajectory: (a) the current joint angles; and (b) the approach vector ***pv***. To address these factors, two pivotal coefficients $\xi^{\left(k\right)}$ and $\delta^{\left(k\right)}$ are introduced; $\xi^{\left(k\right)}$ and $\delta^{\left(k\right)}$ are shown as Equations (23) and (24), respectively:

(23) $$\xi^{\left(k\right)}=\sqrt{1-{\left(\varphi^{\left(k\right)}\right)}^{2}}$$

where $\varphi^{\left(k\right)}=\left({\theta^{\left(k\right)}}_{max}-\theta^{\left(k\right)}\right)/\left({\theta^{\left(k\right)}}_{max}-{\theta_{min}}^{\left(k\right)}\right)$, and $\xi^{\left(k\right)}$ is used to control the increment of joint angles at the next iteration (e.g., as the joint angle increases, the angular velocity should be slower when the angle value approaches the threshold).

(24) $$\delta^{\left(k\right)}=∥pv_{i}∥\left(1-\exp\left(-\eta \frac{∥pv∥}{∥pv_{i}∥}-\gamma \right)\right)$$

$\delta^{\left(k\right)}$ denotes the influence of the approach vector ***pv*** on the next point of the trajectory. A smaller step should be taken when the end-effector is closer to the target at the next moment; *η* and *γ* are coefficients to be calibrated. With this implementation, the end-effector can achieve a stable and smooth trajectory. When the end-effector is moving, the proposed method provides a wide range of speeds, and even makes a full stop if necessary. After considering the influence of the current joint state, Equation (22) needs to be changed into Equation (25), as follows:

(25) $$\underset{\Delta \theta^{\left(k\right)}}{arg}\;\;\;max{\left(\left[\sum \left(∥c_{k}∥\cdot \frac{\Delta \theta^{\left(k\right)}}{\xi^{\left(k\right)}\cdot \delta^{\left(k\right)}}\cdot sin\vartheta^{\left(k\right)}\right),\sum \left(∥c_{k}∥\cdot \frac{\Delta \theta^{\left(k\right)}}{\xi^{\left(k\right)}\cdot \delta^{\left(k\right)}}\cdot cos\vartheta^{\left(k\right)}\right)\right]\cdot pv\right)}^{2}$$

Equation (25) is a convex function. The angle increment of each joint $\Delta \theta^{\left(k\right)}$ is obtained by solving Equation (25), and then is described in the coordinate system. After Equation (25) is solved, the polynomial function given in Equation (4) is used to fit these points. Considering the impacts of the joint angles, the link vectors, the approach vector, and the parameters defined in Equations (23) and (24), in the proposed method, the pseudo-code describing the proposed improved trajectory algorithm (Algorithm 1) is presented as follows:
  **Algorithm 1:** Trajectory Planning   
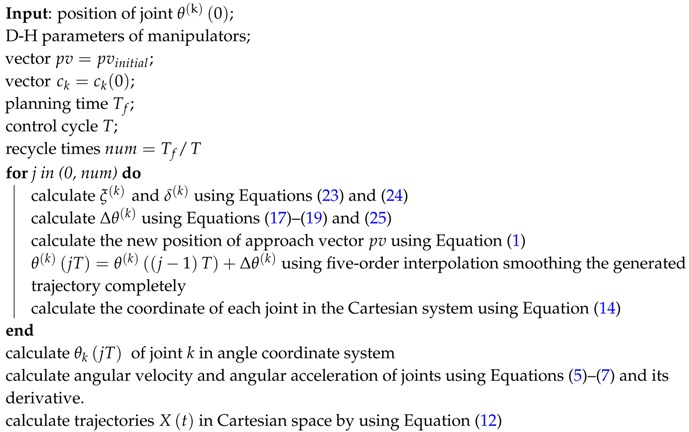



At each iteration, $\theta^{\left(k\right)}\left(jT\right)$ is changing with a controller that makes the angle follow the desired angle increment rate. In this way, an adapted angle increment that is varied with the current condition can be obtained.

## 5. Experiments and Analysis

### 5.1. Experiment Environment

**Figure 6:**
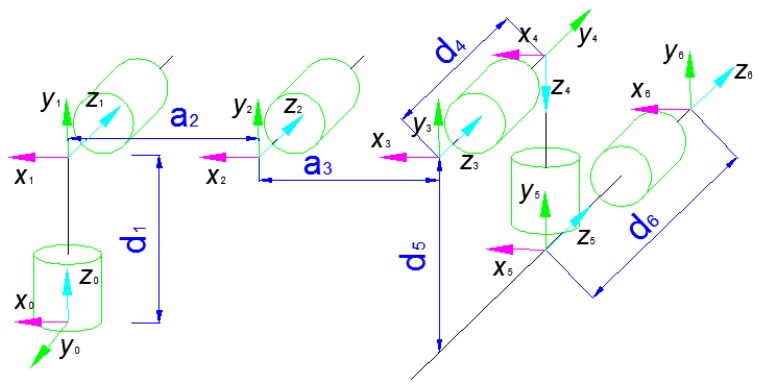
Robotic kinematic model of UR5.

The proposed trajectory planning method is tested on the UR5 robot with 6-DOF. Figure 6 shows the kinematic model, and Table 1 gives the D-H parameters of UR5.

According to the D-H parameters of the UR5 robot, the transformation ${}_{k-1}^{k}A$ from link *k* to k−1 can be derived. Therefore, the vectors described in Figure 5 can be obtained by

(26) $$\left\{\begin{array}{c} b_{k}=b_{k-1}\;\;\cdot {}_{k-1}^{k}A\\ c_{k}=b_{k}+c_{k+1} \end{array}\right.\;$$

Supposing k=6 and $c_{k}=b_{k}$, the Cartesian coordinates of the object $p_{target}$ and the end-effector $p_{end-effector}$ in the base coordinate system can be obtained by using the left and right cameras’ calibration. The approach vector can be calculated by $pv=p_{target}-p_{end-effector}$. After all unknown parameters are obtained, the real-time trajectory can be calculated by Algorithm 1. The calibration of the camera is divided into two steps. The first step is the calibration of the single camera, and the second step is the stereo calibration of the left and right cameras. The camera’s internal and external parameters can be obtained using the principles of stereo imaging and camera calibration described in Section 3. After completing the stereo calibration, the relative position between the left and right cameras will not change; otherwise, the left and right cameras will need to be calibrated again. The Cartesian coordinate of the target object in the world coordinate system is usually calculated by analyzing the visual disparity between the left and right image planes.

### 5.2. Experimental Results

**Figure 7:**
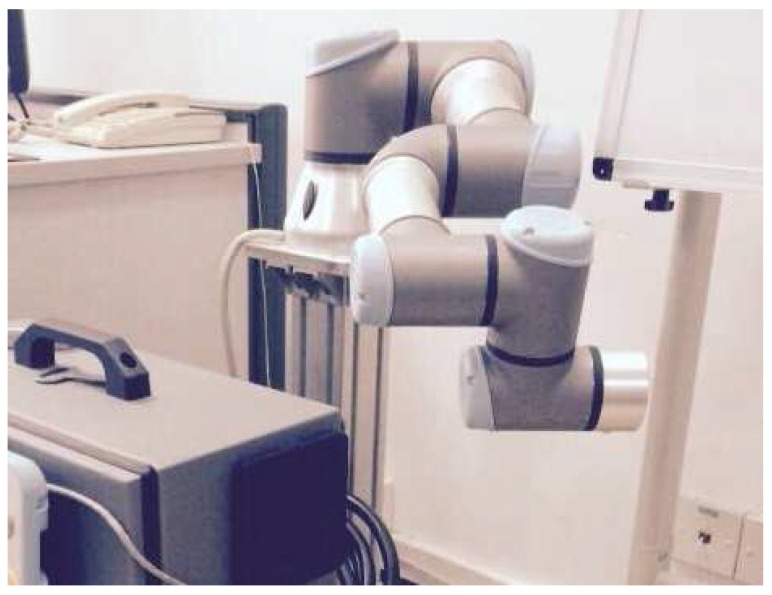
UR5 robot used in the experiments.

In order to verify the effectiveness of our approach, we compare this method with the time optimal algorithm [21]. The UR5 robotic—as shown in Figure 7—is controlled to reach the same target position from the same starting state in these two methods, respectively. Table 2 shows the initial state and the terminate states of the joint angles. In the simulation, the rotation of the sixth joint has little impact on the position of the end-effector. Therefore, its motion is ignored.

**Figure 8:**
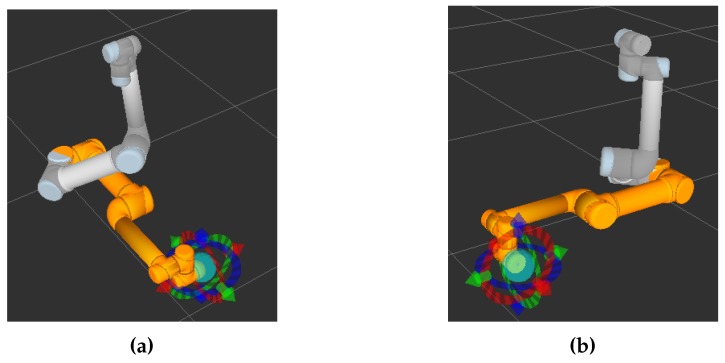
Robot state. (**a**) The initial and terminate state; (**b**) The state from another perspective.

**Figure 9:**
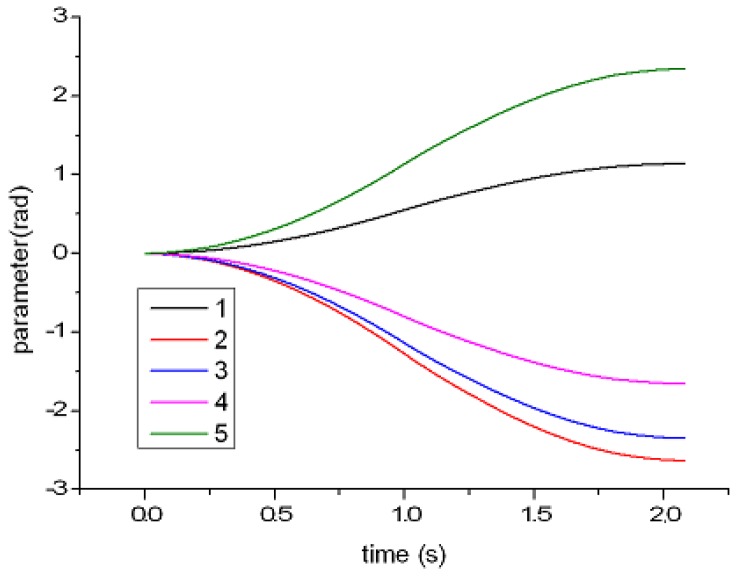
The angle variation of each joint.

**Figure 10:**
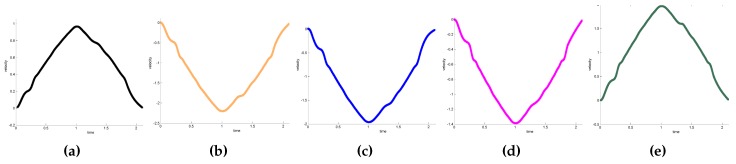
The angular velocity. (**a**) 1st joint; (**b**) 2nd joint; (**c**) 3rd joint; (**d**) 4th joint; (**e**) 5th joint.

The trajectories are recorded to compare these two methods. Figure 8 shows the initial and final states of UR5 at different viewing angles. The initial and final states are indicated by yellow and gray, respectively. Figure 9 shows the variation of each joint angle during the motion. Figure 10 shows the angular velocity of each joint. All angular velocities are less then 3.14 rad/s. Figure 10a–e represent the trajectories of the first, second, third, fourth, and fifth joint, respectively. As shown in Figure 9 and Figure 10, both the angular velocity and angle variation show smooth curves with the proposed trajectory planning method.

**Figure 11:**
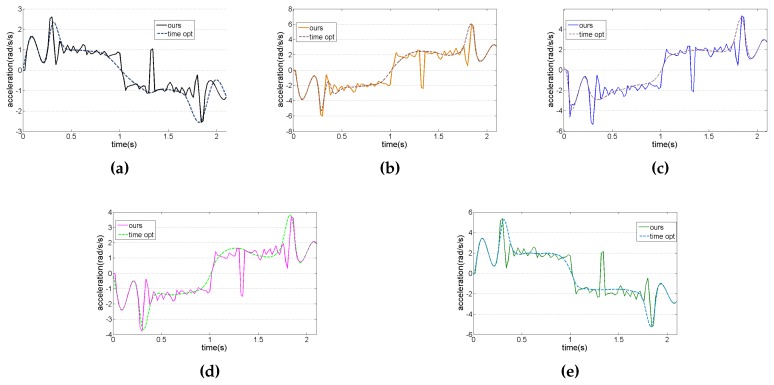
The acceleration curve. (**a**) 1st joint; (**b**) 2nd joint; (**c**) 3rd joint; (**d**) 4th joint; (**e**) 5th joint.

**Figure 12:**
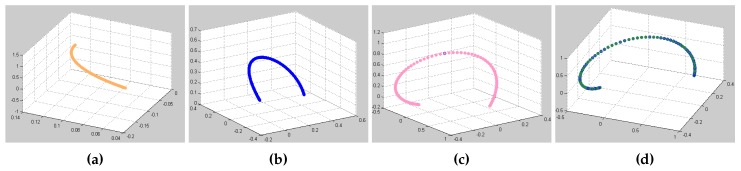
Corresponding trajectories. (**a**) 2nd joint; (**b**) 3rd joint; (**c**) 4th joint; (**d**) 5th joint.

Figure 11 shows the acceleration curve of each joint. The dotted line indicates the time-optimal method. The solid line indicates the acceleration curve of the proposed method, which is controlled and adjusted by the visual system. These solid lines are fitted with the smoothing process of fifth-order interpolation. In this experiment, sampling time is 20 ms, and the operation time of the proposed method and time-optimal method are 2.1 and 1.9 s, respectively. For better comparison, the operation time of the time-optimal method is extended from 1.9 s to 2.1 s in Figure 11. Figure 12 shows the trajectory calculated by Equation (11).

Although the time-optimal method is faster than the proposed method by 0.2 s, there is always some offset between the final position of the end-effector and the target object, which is caused by the errors of mechanical movement and the camera calibration. The proposed method, however, can adjust the robotic motion according to the relative position between the end-effector and the target object to avoid motion and calibration errors, and is therefore able to greatly reduce the position errors. Table 3 shows five records of the comparison test, demonstrating that the proposed method is much better than the time-optimal method at motion precision, which is reflected by the absolute errors.

## 6. Conclusions

This paper presents an effective robotic sensor planning method for CPSS which differs from traditional polynomial interpolation and inverse trajectory planning methods. This method fully considers the positions and conditions of robotic joints. The influences of the joint angles, link vectors, and approach vectors are analyzed to improve planning performance. An optimization function is adopted to generate several intermediate points, which are regressed to a quantic polynomial. Ultimately, a smooth trajectory can be generated for the robotic sensor. Experimental results demonstrate that the proposed method is feasible and effective.

## Figures and Tables

**Table 1:** The parameters of the UR5 robot.

Joints	1	2	3	4	5	6
Torsion angle $\alpha_{k}\;$ (rad)	π/2	0	0	π/2	−π/2	0
Rod length $a_{k}\;$ (mm)	0	–425	–392	0	0	0
Bias length $d_{k}\;$ (mm)	89.2	0	0	109.3	94.75	82.5
Joint angle $\theta^{\left(k\right)}$	$\theta^{\left(1\right)}$	$\theta^{\left(2\right)}$	$\theta^{\left(3\right)}$	$\theta^{\left(4\right)}$	$\theta^{\left(5\right)}$	$\theta^{\left(6\right)}$
Constraint of joint (rad)	±π/2	±π/2	±π/2	±π/2	±π/2	±π/2

**Table 2:** The initial and terminate joint angles of UR5 robot in the experiment.

*θ*	Initial State	Terminate State
1	0	1.142958
2	0	–2.630475
3	0	–2.346571
4	0	–1.654041
5	0	2.346625
6	0	0

**Table 3:** The end-point errors of the UR5 robot (unit: mm).

No.	Coordinates of Target	Final Position Coordinate (Time-Optimal/Ours)	Absolute Errors (Time-Optimal/Ours)
1	(328.04,115.59,341.21)	(336.34,124.31,351.14); (329.73,117.62,342.67)	15.63; 3.02
2	(349.76,273.16,345.68)	(354.13,274.89,349.98); (361.22,275.72,346.12)	6.68; 2.97
3	(401.58,178.29,323.17)	(407.19,183.62,324.55); (401.77,180.37,323.98)	7.87; 2.24
4	(345.43,242.75,350.48)	(352.04,247.70,356.78); (345.54,244.85,352.44)	10.39; 2.87
5	(327.12,–41.74,301.31)	(332.79,–34.30,303.20); (328.80,–41.57,301.83)	9.54; 1.77
